# Ammunition Ship Explosions in Papua New Guinea and Solomon Islands, 1944 and 1945

**Published:** 2025-04-20

**Authors:** G. Dennis Shanks

**Affiliations:** Australian Defence Force Infectious Disease and Malaria Institute, Gallipoli Barracks, Enoggera, Queensland and University of Queensland, School of Public Health, Brisbane, Herston

**FIGURE.**
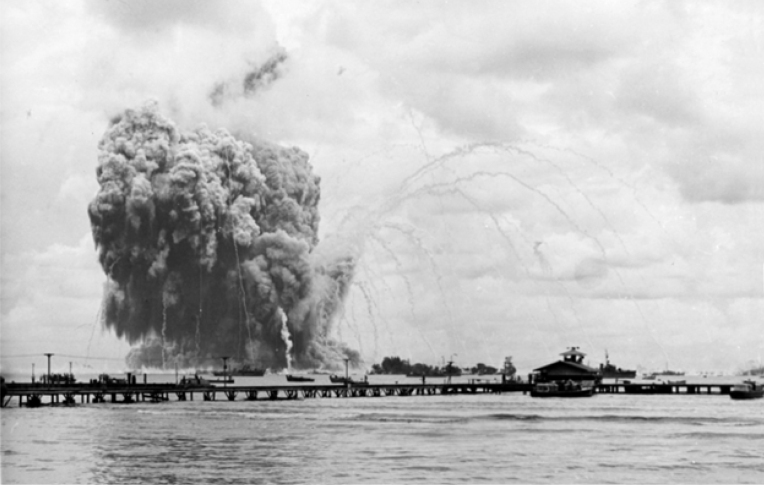
Explosion of *USS Mount Hood* (AE-11) in Seeadler Harbor off Manus Island in Papua New Guinea, November 10, 1944

Seeadler (Sea Eagle) Harbor on the island of Manus in Papua New Guinea was a vital logistics hub for the invasion of the Philippines during the Second World War. The USS *Mount Hood* (AE-11) was unloading munitions from all 5 holds into landing crafts medium (LCMs) while at anchor in the harbor center in November 1944. The ship suddenly exploded on November 10, 1944. ^1^ The blast involved more than 3,800 tons of munitions and killed all 350 on board ship and surrounding LCMs, in addition to 82 crew members on the *USS Mindanao* (ARG-3)—over 300 meters away. No identifiable human remains were recovered from the *Mount Hood* . An additional 371 men were wounded.

The largest piece of the *Mount Hood* 's wreckage located was 30 meters long, sub-merged in a 26 meter-deep crater in the reef. Twenty-two other ships or landing craft were either sunk or severely damaged by the blast. Subsequent investigation concluded “the most likely cause of the explosion was careless handling of ammunition.”

Mishandling military explosives and ammunition has a long history of causing mass casualties. Ammunition ships were particularly high-risk environments for their crews, especially during the laborious process of transferring inherently hazardous explosives. The destruction of ammunition ships in the Indo-Pacific region during the Second World War are only marginally part of our military history as their losses were actively suppressed due to wartime concerns about security and morale.

Just over 2 months after the explosion of the *Mount Hood* , the ammunition ship USS *Serpens* (AK-97) exploded, on January 29, 1945, while loading depth charges off Lunga Point, near Honiara, Solomon Islands. The casualties of that explosion included 250 U.S. Coast Guard crew, Army stevedores, and a medical officer. Two crew on the ship survived the blast in a bow section that continued to float temporarily after the blast.

Although the cause of the *Serpens* explosion remained unclear, the U.S. Navy noted that the loss was not due to enemy action but an “accident intrinsic to the loading process.” The explosion of the USS *Serpens* remains the greatest single mortality event in the history of the U.S. Coast Guard and is marked by a mass grave and monument in the Arlington National Cemetery. ^2^

These accidental ship explosions during the Second World War caused mass casualties without any enemy intervention. Lessons were uncertain and indefinite, as any forensic evidence was destroyed by the blast wave. Wartime secrecy as well as bureaucratic disinclination for admitting failure has made these accidents much less well-known then when the same munitions were used by troops to defeat Imperial Japan. ^3^

Caution with ammunition is always indicated, but recent events, particularly with explosions at ammunition depots in the developing world—Lagos in 2002, Maputo in 2007, and Brazzaville in 2012—should serve as an important reminder that weapons have the potential to kill friend and foe alike if mishandled. Ammunition is both a disarmament as well as a public health danger that requires unremitting vigilance.

The author, of both *Images in Health Surveillance* featured in this issue, acknowledges the service and sacrifice of all those who served in the military during the Second World War and thanks the many unnamed military officers, scientists, historians, and medical librarians who have unselfishly provided data and ideas for these manuscripts, especially the librarians at the Australian Defence Force Library at Gallipoli Barracks, Queensland.
